# Unmeasurable capillary C-reactive protein as one of the diagnostic clues of severe hematological pathologies in children in primary settings: Case series

**DOI:** 10.1097/MD.0000000000035776

**Published:** 2023-10-27

**Authors:** Barbora Piteková, Jakub Zieg, Patrik Konopásek, Ladislav Turecký, Marcel Brenner, Jakub Gécz

**Affiliations:** a Department of Pediatric Emergency Medicine, National Institute of Children’s Diseases, Bratislava, Slovakia; b Department of Pediatric Urology, Faculty of Medicine, Comenius University and National Institute of Children’s Diseases, Bratislava, Slovakia; c Pediatric Department, 2nd Faculty of Medicine, University Hospital Motol, Charles University, Prague, Czech Republic; d Department of Medical Chemistry, Biochemistry and Clinical Biochemistry, Medical Faculty, Comenius University, Bratislava, Slovakia.

**Keywords:** acute leukemia, children, chronic leukemia, C-reactive protein, fever

## Abstract

**Rationale:**

The manuscript aimed to show that an unmeasurable capillary C-reactive protein (CRP) should be a red flag that can indicate a possible severe hematological pathology.

**Patients concerns and diagnoses:**

The authors present 3 case reports of children with fever examined at the pediatric emergency department. Fever is among the most frequently exhibited symptoms of acute pediatric infectious diseases. However, sometimes fever can be the manifestation of other serious noninfectious diseases. CRP is a marker widely used in clinical pediatric practice to help us evaluate inflammation and possible bacterial infection. All mentioned patients had unmeasurable CRP from capillary blood, even though venous CRP ranged from 14 to 21 mg/L. All of the patients were consequently diagnosed with severe hemato-oncological disease. Possible explanations are that a change in blood viscosity or an elevation of circulating immune complexes in the blood of patients with leukemia leads to malfunctioning immunoturbidimetry measurement.

**Lesson:**

Although these findings are very interesting and could lead to faster recognition of acute leukemia in pediatric clinical practice, further prospective study is needed for their confirmation.

## 1. Introduction

Leukemias are the most common oncological diseases in the pediatric population. However, because of their often nonspecific clinical symptoms in their early stages similar to common viral infections, they can represent a diagnostic dilemma in primary clinical settings.^[1]^ This can delay the diagnosis of a malignancy, which affects the morbidity and mortality of such patients.^[2]^ C-reactive protein (CRP) is an inflammatory marker widely used in clinical pediatric practice in children with fever to help to decide if infection is viral or bacterial.^[3,4]^ Measuring CRP has limitations and test results can be influenced by several factors.^[5]^ The authors present 3 case reports from their own clinical practice, in which the basal examination of capillary CRP played an unusual, but important role, together with more complex blood samplings to reach a prompt and correct diagnosis of a binding onco-hematological disease in the patients.

## 2. Case report 1

The first case involved a 13-year-old female who came to the Department of Pediatric Emergency Medicine with a fever 40.0 °C and symptoms of an acute respiratory tract infection. On physical examination, the patient was subfebrile, with tachycardia (144/min), with signs of incipient tonsillopharyngitis and splenomegaly (+3 cm bellow the costal margin). The patient was suspected of having an incipient acute mononucleosis and CRP examination from capillary blood was performed to distinguish between viral and bacterial infection. The result of the examination was repeatedly unmeasurable (high blank signaled error). Because of this, venous sampling was performed on the patient, including blood count and inflammatory parameters. Blood count revealed hyperleukocytosis with neutrophilia (white blood count [WBC] 421 × 10^9^/L, neutrophils 374 × 10^9^/L), moderate anemia (hemoglobin 82 g/L), and normal platelet count (thrombocytes [Tr] 191 × 10^9^/L). CRP taken from peripheral blood was only mildly elevated (21.3 mg/L), procalcitonin was in the reference range (0.3 ug/L). Bone marrow puncture and flow cytometry revealed the significant granulocytic proliferation. Cytogenetically, the translocation of chromosomes 9 and 22 was confirmed and the diagnosis was concluded as chronic myeloid leukemia (CML), Ph + positive.

## 3. Case report 2

The second case was a 6-year-old male who was examined by his general practitioner for a 2 day fever and the occurrence of petechiae on the skin. CRP examination from capillary blood was carried out, repeatedly with an unmeasurable result (low hematocrit signaled error). The patient was sent to the Department of Pediatric Emergency Medicine in our hospital due to suspected meningococcal meningitis. Physical examination revealed an anemic syndrome (tachycardia 115/min, skin pallor), splenomegaly (+5 cm bellow the costal margin), and numerous small petechiae on the chest and lower limbs while, meningeal signs were negative. Blood count showed leukocytosis with lymphocytosis (WBC 178 × 10^9^/l, lymphocytes 156 × 10^9^/l), severe anemia (hemoglobin 37 g/l), and severe thrombocytopenia (Tr 9 × 10^9^/l). CRP taken from the peripheral blood was slightly elevated (17 mg/l) procalcitonin in reference range (0.19 ug/l). More than 84% of pathological cells with the expression of CD10+, CD19+, and CD20 signs were found in the patient’s peripheral blood. Based on these results the patient was diagnosed with acute B-lymphoblastic leukemia, which was subsequently confirmed by bone marrow aspiration.

## 4. Case report 3

The third case involved a 6-year-old male who was examined at the pediatric emergency service for cervical lymphadenopathy. The patient had a short 24-hour fever, fatigue, and frontal headache without signs of an acute respiratory infection. The parents noticed enlarged lymph nodes on the right side of the neck. The pediatrician at the emergency service examined the CRP with an unmeasurable result (low hematocrit signaled error) and the patient was sent to the Department of Pediatric Emergency Medicine. Based on our previous experience with unmeasurable capillary CRPs and the clinical signs, serious illness was suspected and we immediately performed complete venous sampling including a blood count on the patient. During the physical examination, bilateral cervical lymph nodes (submandibular, along the sternocleidomastoid muscle) were palpable, elastic and freely mobile, size 2 × 1 cm. In addition, the supraclavicular lymph nodes on both sides were also palpable, other lymph nodes were not enlarged, and the liver and the spleen were not palpable. We observed a couple of small hematomas on the lower limbs. Blood count revealed leukocytosis with lymphocytosis (WBC 325 × 10^9^/L, lymphocytes 289 × 10^6^/L), moderate anemia (hemoglobin 81 g/L), and thrombocytopenia (Tr 64 × 10^9^/L). Inflammatory parameters taken from the peripheral blood were slightly elevated (CRP 14 mg/L, procalcitonin 0.55 ug/L). Chest X-ray showed significant mediastinal lymphadenopathy. More than 89% of pathological T-lymphoid cells were found in the patient’s peripheral blood. Based on these results the patient was diagnosed with acute T lymphoblastic leukemia, which was confirmed by bone marrow aspiration.

## 5. Discussion

In general, oncologic diseases in children are rare, but acute leukemia is the most frequent form of childhood cancer (representing in Europe from 25% to 30% of all oncologic disease, depending on age at the time of diagnosis).^[6]^ The incidence of acute leukemia in childhood is approximately 4 cases per 100,000 children.^[7,8]^ The highest incidence is between 2 to 5 years of age, more often in boys.^[7]^ The basic pathophysiological mechanism is that abnormal hematopoietic cells start their unregulated clonal proliferation, resulting in bone marrow failure. In pediatrics, we mainly encounter acute lymphoblastic leukemia (ALL) and acute myeloid leukemia, but rarely will clinicians see CML.^[9]^ Typical clinical symptoms of acute leukemia arise from bone marrow infiltration and extramedullary involvement. Patients can present with anemic syndrome (fatigue, pallor), neutropenia (infection), thrombocytopenia (petechia, purpura), and also with extramedullary manifestation such as lymphadenopathy, hepatomegaly, and splenomegaly. However, all these symptoms are insignificant and nonspecific and can imitate many other benign diseases, such as infectious mononucleosis or other acute viral infections. Approximately half of the patients with acute leukemia initially exhibit a fever from released pyrogenic toxins from leukemic cells. It can cause a diagnostic dilemma for the primary care pediatrician and may be difficult to distinguish from ordinary childhood diseases.^[1,10]^ The majority of patients diagnosed with CML are asymptomatic, when symptoms do occur, it is the result of anemia and splenomegaly.^[11]^ Rigorous study of medical history and physical examination are fundamental in diagnosing leukemia. The next step in diagnostic assessment is a blood count examination. Leukocytosis and a decrease in other blood components are common in children with leukemia, but some children can also exhibit low or normal leukocyte values. However, nonspecific symptoms can delay the performance of appropriate blood tests and a subsequent correct diagnosis. Delayed diagnosis of acute leukemia is associated with increased mortality and morbidity.^[2]^

CRP is synthesized predominantly in the liver, after induction of IL-6 and other inflammatory cytokines. Its increased production is a response to the inflammatory process in the body. It has an essential function in the recognition, binding, and clearance of pathogens and infected cells.^[3]^ This test is indicated when an infection or chronic inflammatory diseases such as lupus or rheumatoid arthritis are suspected. In acute infection, CRP is a useful tool to distinguish between a bacterial or viral cause of disease. Concentrations of CRP start to increase in serum approximately 6 to 12 hours with maximum concentration after 24 to 72 hours after the onset of a bacterial infection. A quantitative CRP point-of-care test can be performed using venous blood, or, especially, in a primary care setting quickly and easily, by examining capillary blood samples. The capillary CRP test has a good correlation with the standard biochemical determination of CRP from venous blood.^[4,12]^ The CRP value serves as an auxiliary examination for pediatricians in children with a fever and signs of an acute infection, when deciding on antibiotic treatment.^[3,4]^ In our conditions, the QuikRead Go CRP device (a product of Orion, Finland) is freely used to determine capillary CRP values, where the determination is carried out by the immunoturbidimetry method. Immunoturbidimetry is a measurement of light by a spectrophotometer that remains after the incident light has been absorbed by the immune complexes in the solution in the cuvet to determine concentration of particles in a sample. The correct measurement of this method can be influenced by device related and non-device related technical issues. Non-device related factors are the concentration of the solution used, and the temperature and viscosity of the blood in the sample.^[4,5]^ The result is available in 2 minutes and is reported between <5 and 200 mg/L. Each examination of CRP in the mentioned patients was carried out from capillary blood and on this device.^[5]^ In 2022 we performed 1370 examinations using the QuikRead Go CRP with the exact results except in the first mentioned case. The first possible explanation is that the changed blood viscosity, either increased in the case of significant leukocytosis or decreased in the case of severe anemia or thrombocytopenia, makes it impossible for the device to correctly measure the level of CRP.^[5]^ The second possible explanation is that patients with acute or chronic leukemia have increased circulating immune complexes in the blood, which could precipitate in the solution and immunoturbidimetry measurement could not work properly.^[13]^ These factors could lead to an unmeasurable result and the above mentioned errors such as high blank or low hematocrit.

In the 3 case reports of pediatric patients who showed up with a short lasting fever, we tried to demonstrate how the commonly used parameter, capillary C-reactive protein, was one of the clues to reach the correct diagnosis in these patients. In all patients, unmeasurable CRP was assessed in capillary blood, venous inflammatory parameters were just slightly elevated (ranging from 14 to 21 mg/L), and severe hemato-oncological disease was confirmed (one patient was diagnosed with CML, the second with B-ALL, and the third with T-ALL). Based on our knowledge, a similar work has not yet been published. The strongest limitation of our paper is that we only described 3 case reports. Although, our observation is very interesting and could possibly lead to faster diagnosis of acute leukemia, for their implementation in daily pediatric clinical practice further prospective study with a cohort group of patients is needed.

In conclusion, these findings are very valuable not only for pediatricians in the pediatric emergency department, but especially for pediatricians working in primary care settings with limited laboratory testing options where capillary CRP is sometimes the only diagnostic tool at their disposal. The result of an unmeasurable capillary CRP should be a red flag which can indicate a possible severe hematological pathology in patients.

## Acknowledgments

We would like to thank Noemi Madarasz M.D., for diagnosis of the third patient, the pediatric hematooncologist Karel Svojgr M.D., PhD. and the clinical biochemist Filip Micev M.D. for critical review of the manuscript.

## Author contributions

**Formal analysis:** Barbora Piteková, Jakub Zieg, Patrik Konopásek, Jakub Gécz, Marcel Brenner.

**Funding acquisition:** Barbora Piteková.

**Investigation:** Barbora Piteková, Jakub Zieg, Ladislav Turecký, Jakub Gécz.

**Supervision:** Jakub Zieg, Jakub Gécz.

**Writing – original draft:** Barbora Piteková.

**Writing – review & editing:** Barbora Piteková, Jakub Zieg, Patrik Konopásek, Ladislav Turecký, Jakub Gécz, Marcel Brenner.
